# Sex-related hormonal variances and clinical outcomes in TAVR patients

**DOI:** 10.1007/s00392-025-02623-6

**Published:** 2025-02-24

**Authors:** Mustafa Mousa Basha, Baravan Al-Kassou, Marcel Weber, Thomas Beiert, Farhad Bakhtiary, Sebastian Zimmer, Georg Nickenig, Philip Roger Goody, Jasmin Shamekhi

**Affiliations:** 1 Heart Center, Department of Medicine II, University Hospital Bonn, Venusberg-Campus 1, 53127 Bonn, Germany; 2 Heart Center Bonn, Department of Cardiac Surgery, University Hospital Bonn, Bonn, Germany

**Keywords:** Aortic stenosis, Hormonal factors, Estradiol, Testosterone, Parathormone, Cortisol, IGF-1, DHEAs, Transcatheter Aortic Valve Replacement (TAVR), Clinical outcomes

## Abstract

**Background:**

Sex-related differences play a pivotal role in disease manifestation and outcome in patients with cardiovascular disease, including aortic valve stenosis (AS). However, data regarding sex-related hormonal differences in AS patients undergoing transcatheter aortic valve replacement (TAVR) is lacking.

**Objectives:**

We aimed to assess sex-related hormonal variances in patients with severe symptomatic AS and to evaluate the impact of these hormonal differences on the clinical outcomes after TAVR.

**Methods:**

In a total of 361 TAVR patients, we assessed the hormonal status, including cortisol, parathormone (PTH), insulin-like growth factor 1 (IGF-1), dehydroepiandrosterone sulfate (DHEAs), estradiol, progesterone and testosterone prior to TAVR. We compared baseline characteristics and outcome data according to sex and hormonal parameters. The primary endpoint was 1-year all-cause mortality according to sex; secondary endpoints included the risk of 1-year all-cause mortality in conjunction with hormone levels, with pre-specified cut-off values.

**Results:**

Rates of 1-year all-cause mortality were comparable between the sexes (p = 0.285). Cox regression analysis revealed significant associations between 1-year mortality and levels of cortisol (HR 2.30; p = 0.007), PTH (HR 2.09; p = 0.019), DHEA-S (HR 0.47; p = 0.016), and IGF-1 (HR 0.42; p = 0.004) in the overall cohort. Elevated cortisol levels (p = 0.011), decreased DHEA-S levels (p = 0.007), and lower IGF-1 levels (p = 0.017) were significantly associated with higher rates of 1-year all-cause mortality in males. Conversely, higher PTH levels were significantly associated with an increased risk of 1-year mortality in females (p = 0.012).

**Conclusion:**

Sex-specific hormonal differences significantly impact the prognosis of severe AS patients undergoing TAVR. Elevated cortisol levels and decreased DHEA-S and IGF-1 levels in males, as well as higher levels of PTH in females, were associated with an increased mortality risk.

## Introduction

Aortic valve stenosis (AS) is one of the most common valvular heart disorders and the prevalence is expected to further increase as the population ages. Once symptomatic, patients with severe AS have significantly increased rates of morbidity and mortality [1, 2].

The pathobiology of degenerative AS is complex and involves multiple mechanisms, including induction of valve fibrosis, inflammation, oxidative stress, angiogenesis, hemorrhage, and osteogenic differentiation [3]. Interestingly, sex distribution differs across age subgroups. Women represent the majority of patients with severe AS over 80 years of age [4] and there are increasingly recognized pathophysiological differences between men and women with AS. These involve the role of aortic valvular calcifications in relation to AS severity [5] and the left ventricular (LV) response to AS hemodynamic burden [6].

Many studies have already demonstrated the effects of various hormones, including sex hormones, parathyroid hormone (PTH), and cortisol, on the progression of AS and cardiovascular risk, suggesting that some hormonal changes might be associated with an increased mortality risk [7–9]. Conversely, previous data have shown that dehydroepiandrosterone sulfate (DHEA-S) and insulin-like growth factor 1 (IGF-1) may play a protective role in the development of atherosclerosis and cardiovascular events [10].

In this study, we hypothesize that the hormonal status of older patients, particularly cortisol, PTH, DHEA-S, and IGF-1 levels, influences the outcomes of patients with AS after valve repair, with different effects observed between sexes. To address these effects, we aimed to assess the hormonal status in patients with severe symptomatic AS and to evaluate the impact of hormonal differences on clinical outcomes after transcatheter aortic valve replacement (TAVR), while considering sex-related differences.

## Methods

### Patient population and hormonal parameters

We retrospectively analyzed 361 patients with severe symptomatic AS and increased surgical risk who underwent TAVR between January 2018 and April 2019 at the Heart Center Bonn. The retrospective use of patients’ data for scientific needs was approved by the local ethics committee and written informed consent was obtained from all patients prior to the procedure.

Before the TAVR procedure, all patients underwent a careful evaluation including pre-interventional transthoracic and transesophageal echocardiography (including three-dimensional measurements) and had an interdisciplinary discussion with the local institutional Heart Team. Details concerning patient screening, procedural techniques, and adjunctive medication have been described elsewhere [11, 12].

In this study, we included all patients whose hormonal status was assessed prior to TAVR. The hormonal tests included cortisol, PTH, IGF-1, DHEA-S, estradiol, progesterone, and testosterone to evaluate sex-related differences. No additional hormonal markers were assessed.

### Study endpoints

The primary endpoint of the study was 1-year all-cause mortality according to sex. Additionally, we assessed secondary endpoints, including the risk of 1-year all-cause mortality in accordance with hormone levels and sex-related differences.

### Statistical analysis

Normally distributed data are shown as mean ± standard deviation or as counts with percentages, non-normally distributed data is presented as a median with range or inter-quartile range (IQR). P values have been used for comparison using student’s t-test, Fisher exact tests or chi-squared tests as appropriate. A p value of < 0.05 was deemed significant. The log-rank test was used to determine statistical differences in terms of survival. Individual risk factors were evaluated using logistic regression, estimating hazard ratios (HRs) and corresponding 95% confidence intervals.

We compared baseline characteristics and the hormonal status between sexes, and focused on the association between the hormonal status and the clinical outcome after TAVR.

Additionally, we performed a Cox regression analysis for 1-year all-cause mortality in accordance with hormonal cut-off values and sex. The hormonal cut-off values were determined using the overall median; for testosterone, sex-related medians were used. The cut-off values were as follows: PTH > 75 pg/ml vs. PTH: ≤ 75 pg/ml, DHEA-S > 0.45 µg/ml vs. DHEA-S ≤ 0.45 µg/ml, IGF-1 > 72 ng/ml vs. IGF-1 ≤ 72 ng/ml, cortisol > 8.4 µg/dl vs. cortisol ≤ 8.4 µg/dl, estradiol > 13 pg/ml vs. estradiol ≤ 13 pg/ml, progesteron > 0.05 ng/ml vs. progesteron ≤ 0.05 ng/ml, testosteron > 0.075 ng/ml vs. testosteron ≤ 0.075 ng/ml, testosteron > 2.82 ng/ml vs. testosteron ≤ 2.82 ng/ml in males.

Statistical significance was assumed when the null hypothesis could be rejected at p < 0.05. Statistical analyses were conducted with SPSS Statistics version 29.0 (IBM Corporation, Somers, NY, USA). The investigators initiated the study, had full access to the data, and wrote the manuscript. All authors vouch for the data and its analysis.

## Results

A total of 361 TAVR patients were included in the present analysis. The patient cohort presented with a mean age of 80.2 ± 7.3 years and an intermediate operative risk (EuroSCORE II: 5.0 ± 5.1; STS-PROM: 4.3 ± 4.0). Approximately half of the patients were male (54%) and all patients underwent transfemoral TAVR. Follow-up outcome data were collected for all 361 patients, with a mean follow-up duration of 342 ± 159 days (median: 365 days; IQR: 241–470).

### Baseline and procedural characteristics according to sex

Baseline and procedural characteristics are presented in Table 1. In our study cohort, women were significantly older than men (81.4 ± 6.6 vs 79.1 ± 7.7; p = 0.004). There were no significant differences according to BMI (p = 0.788), peripheral artery disease (p = 0.487), diabetes mellitus (p = 0.215), NYHA-class IV (p = 0.546), atrial fibrillation (p = 0.284), or NT-proBNP levels (p = 0.549). However, women had a lower prevalence of chronic obstructive pulmonary disease (COPD) (13% vs. 25%; p = 0.003), hypertension (86% vs. 93%; p = 0.037), previous myocardial infarction (16.1 vs. 23.1; p = 0.001), and coronary artery disease (55% vs. 77%; p < 0.001). However, we could not find significant differences with regard to the operative risk between sexes quantified by the EuroSCORE II (5.1 ± 5.0 vs. 5.0 ± 5.0; p = 0.999) and STS-PROM (4.3 ± 4.0 vs. 4.8 ± 4.1; p = 0.050). Procedural characteristics such as the amount of contrast media, the fluoroscopy or procedure time did not differ significantly between women and men.

**Table 1:** Baseline characteristics according to gender

	All patients *n* = 361	Female *n* = 166	Male *n* = 195	*p* value
Age, ± SD	80.2 ± 7.3	81.4 ± 6.6	79.1 ± 7.7	0.004
BMI, ± SD	28.0 ± 11.0	28.1 ± 16	27.8 ± 4.7	0.788
Peripheral artery disease, *n*	137 (38.0)	61 (36.7)	76 (39)	0.487
COPD, *n*	70 (19.4)	21 (13)	49 (25)	0.003
Hypertension, *n*	324 (89.8)	143(86)	181 (93)	0.037
Diabetes, *n*	123 (34.1)	51 (31)	72 (37)	0.215
NYHA IV, *n*	22 (6.1)	12 (7.2)	10 (5.1)	0.546
Previous MI, *n*	58 (16.1)	13 (7.8)	45 (23.1)	< 0.001
Atrial fibrillation, *n*	144 (39.9)	73 (44)	71 (36.4)	0.284
Ejection fraction, %	56.9 ± 12.2	59.5 ± 10	54.8 ± 13	0.001
NT-proBNP, pg/ml	1617 (617/3740)	1464 (715/3588)	1768 (576/3454)	0.549
Coronary artery disease, *n*	242 (67)	92 (55)	150 (77)	< 0.001
AV Pmean, mmHg	40.1 ± 14.1	42.3 ± 15	38.1 ± 12	0.005
AV Vmax, m/s	4.4 ± 0.5	4.7 ± 0.7	4.0 ± 0.6	0.002
AVA, cm^2^	0.75 ± 0.2	0.72 ± 0.2	0.77 ± 0.2	0.024
AVA ≤ 0.5 cm^2^, *n*	56 (15.5)	34 (20.5)	22 (11.3)	0.019
Bicuspid valve, *n*	8 (2.2)	3 (1.8)	5 (2.6)	0.729
AR, *n*	270 (74.8)	120 (72.3)	150 (76.9)	0.322
Severe MR, *n*	21 (5.8)	12 (7.2)	9 (4.6)	0.368
Severe TR, *n*	14 (3.9)	9 (5.4)	5 (2.6)	0.181
sPAP, mmHg	36.0 ± 15.4	36.8 ± 15.7	35.3 ± 15.2	0.443
Euro-Score	16.2 ± 12.4	15.8 ± 11.3	16.5 ± 13.2	0.577
Euro-Score II	5.0 ± 5.1	5.1 ± 5.0	5.0 ± 5.0	0.999
STS-Score	4.3 ± 4.0	4.8 ± 4.1	4.0 ± 3.3	0.050
Contrast media, ml	149.1 ± 192.7	105 ± 56	180 ± 245	0.256
Fluoroscopy time, min	16.4 ± 12.6	13.1 ± 9.7	18.7 ± 14.0	0.195
Procedure time, min	50.7 ± 38.1	41.8 ± 23	57.4 ± 45	0.223

Values are mean (± SD), median (IQR 1/3) or *n*/*N* (%)

*BMI* body mass index, *COPD* chronic obstructive pulmonary disease, *NYHA* New York Heart Association, *MI* myocardial infarction, *NT-proBNP* n-terminal pro brain natriuretic peptide, *AV Pmax* aortic valve maximum pressure, *AV Pmean* aortic valve mean pressure, *AV Vmax* peak aortic valve jet velocity, *AVA* aortic valve area, *AR* aortic regurgitation, *MR* mitral valve regurgitation, *TR* tricuspid valve regurgitation, *sPAP* systolic pulmonary artery pressure, *C-AS* critical aortic stenosis, *EuroSCORE* European System for Cardiac Operative Risk Evaluation, *STS-Score* the Society Thoracic of Surgeons-Score

### Echocardiography

In terms of echocardiographic parameters, women presented with a significantly higher ejection fraction (EF) (56.9 ± 12.2% vs. 54.8 ± 13%; p = 0.001), a higher mean aortic valve pressure gradient (42.3 ± 15 mmHg vs. 28.1 ± 12 mmHg; p = 0.005), a higher maximum aortic valve peak velocity (4.7 ± 0.7 m/sec vs. 4.0 ± 0.6 m/sec; p = 0.002), and a smaller aortic valve area (0.72 ± 0.2 cm^2^ vs. 0.77 ± 0.2 cm^2^; p = 0.024). There were no significant differences regarding accompanying aortic valve regurgitation (p = 0.322), severe mitral valve regurgitation (p = 0.368), severe tricuspid valve regurgitation (p = 0.181), or systolic pulmonary artery pressure (p = 0.443) between the sexes.

### Hormonal parameters

Hormonal parameters according to sex are presented in Table 2. There were no differences between women and men in terms of progesterone (p = 0.269), IGF-1 (p = 0.127), DHEA-S (p = 0.475), or PTH (p = 0.090). As expected, there were significant differences in terms of sexual hormones such as estradiol (female: 8.7 ± 7.5 pg/ml vs. male: 21.0 ± 10.0 pg/ml; p < 0.001), and testosterone (female: 0.2 ± 0.2 ng/ml vs. male: 3.0 ± 1.6 ng/ml; p < 0.001). Interestingly, significant differences were also observed in cortisol levels (female: 10.3 ± 6.1 µg/dl vs. male: 8.4 ± 4.3 µg/dl; p = 0.001).

**Table 2:** Hormonal differences according to gender

	All patients *n* = 361	Female *n* = 166	Male *n* = 195	*p* value
Estradiol, pg/ml	15.4 ± 10.9	8.7 ± 7.5	21.1 ± 10.0	< 0.001
Progesteron, ng/ml	0.07 ± 0.07	0.07 ± 0.07	0.08 ± 0.08	0.269
Testosteron, ng/ml	1.6 ± 1.9	0.2 ± 0.2	3.0 ± 1.6	< 0.001
Cortisol, µg/dl	9.2 ± 5.3	10.3 ± 6.1	8.4 ± 4.3	0.001
Parathormone, pg/ml	95.5 ± 78	103 ± 87	89 ± 69	0.090
IGF-1, ng/ml	102.0 ± 49	97.6 ± 50	105 ± 48	0.127
DHEA-S, µg/ml	0.87 ± 0.5	1.1 ± 7.4	0.7 ± 0.7	0.475

Values are *n*/*N* (%), mean ± SD

### Clinical outcome

Comparing the one-year all-cause mortality rate between sexes, we could not find a significant difference in our study population (female:11.4% vs. male:15.9%; p = 0.285).

In the overall cohort, the cox regression analysis for the risk of 1-year all-cause mortality according to hormone cut-off values revealed statistically significant associations between 1-year mortality and cut-off levels of cortisol (p = 0.007), PTH (p = 0.019), DHEA-S (p = 0.016), and IGF-1 (p = 0.004), Table 3.

**Table 3:** Univariate and multivariate analysis for 1-year mortality in accordance with hormonal cut-offs

	Univariate analysis		Multivariate anaylsis	
Hormones	HR (95% CI)	*p* value	HR (95% CI)	*p* value
Parathormone > 75 pg/ml	2.09 (1.13–3.86)	0.019	1.79 (0.94–3.40)	0.076
DHEA-S > 0.45 µg/ml	0.47 (0.25–0.87)	0.016	0.53 (0.28–1.00)	0.050
IGF-1 > 72 ng/ml	0.42 (0.24–0.76)	0.004	0.54 (0.29–0.98)	0.043
Cortisol > 8.4 µg/dl	2.30 (1.26–4.21)	0.007	1.81 (0.97–3.37)	0.062
Estradiol > 13 pg/ml	1.45	0.212	–	–
Progesteron > 0.05 ng/ml	1.26	0.461	–	–
Testosteron > 2.82 ng/ml	0.531	0.103	–	–

The 95% confidence interval (CI) for the hazard ratio (HR) was only reported when the *p* value was significant (*p* < 0.05)

The analysis indicated that elevated cortisol levels > 8.4 µg/dl were associated with a significantly higher 1-year mortality risk, with an HR of 2.30 with a 95 percent confidence interval of 1.26–4.21 (p = 0.007). PTH levels > 75 pg/ml were also associated with a significantly higher 1-year mortality risk, with an HR of 2.09 (95% CI (1.13–3.86) (p = 0.019)). Conversely, DHEA-S levels > 0.45 µg/ml were associated with a significantly lower risk of 1-year mortality, with an HR of 0.47 (95% CI (0.25–0.87) (p = 0.016)). IGF-1 levels > 72 ng/ml were associated with a lower risk of 1-year mortality, with an HR of 0.42 (95% CI (0.24–0.76) (p = 0.004)). In the multivariate analysis, statistical significance remained for IGF-1 (p = 0.043), while the other hormones showed a trend toward significance but with borderline p-values: PTH (p = 0.076), DHEA-S (p = 0.050), and cortisol (p = 0.062).

**Fig. 1:**
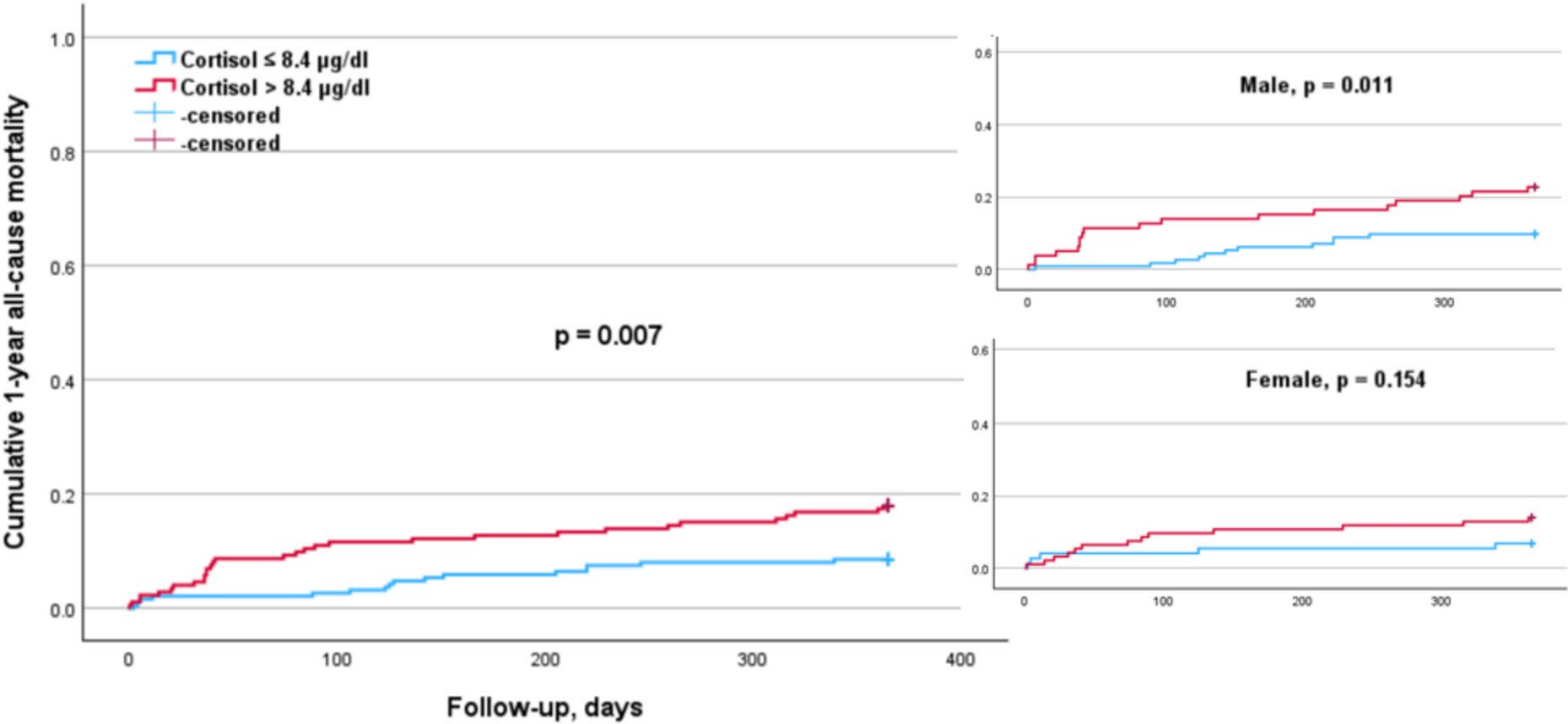
Kaplan–Meier survival analysis of 1-year all-cause mortality according to cortisol levels with a cut-off value of 8.4 µg/dl for the entire cohort, as well as for males and females. The analysis shows significant differences in the overall cohort (*p* = 0.007). This was significant in the male (*p* = 0.011), but not in the female subgroup (*p* = 0.154)

**Fig. 2:**
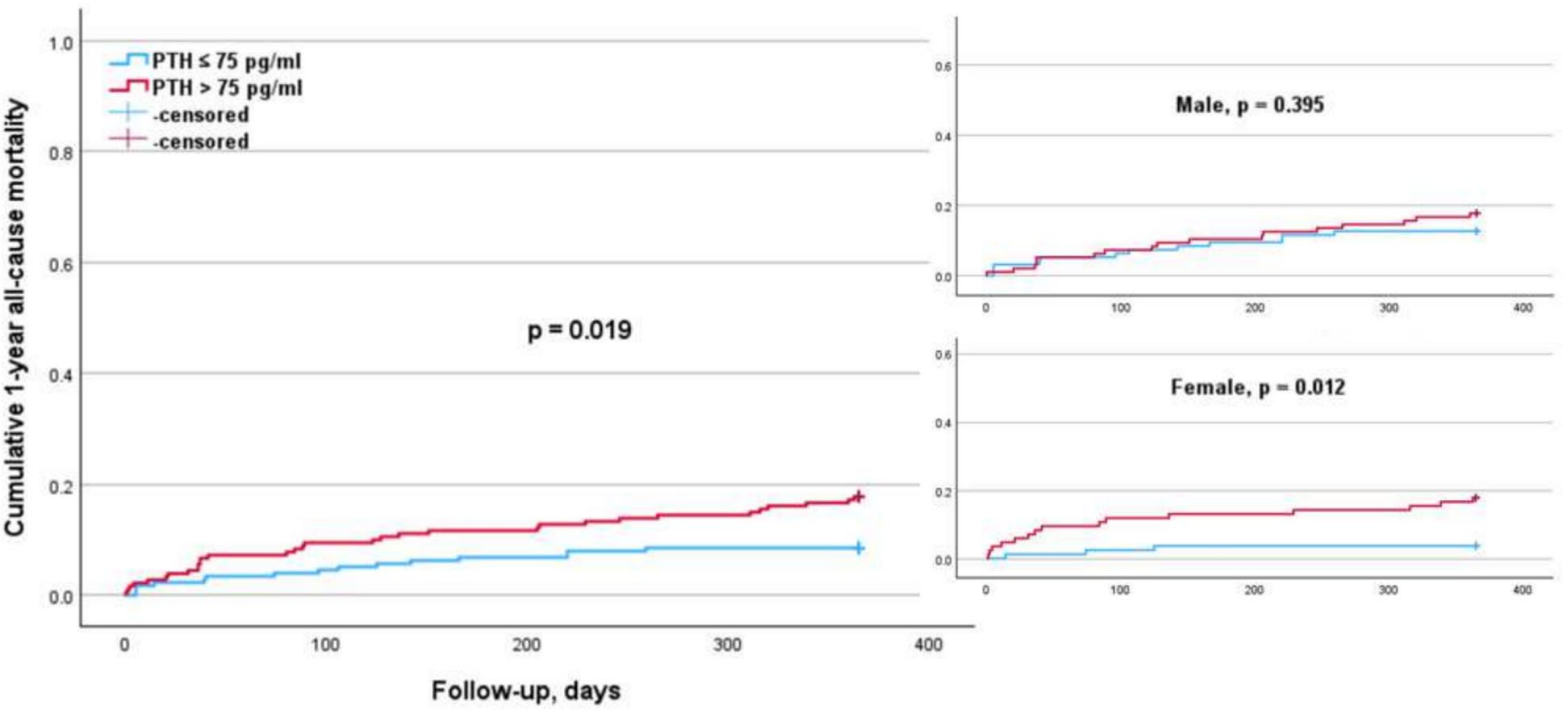
Kaplan–Meier survival analysis of 1-year all-cause mortality according to PTH levels with a cut-off value of 75 pg/ml for the entire cohort, as well as for males and females. The analysis shows significant differences in the overall cohort (*p* = 0.019). This was significant in the female (*p* = 0.012), but not in the male subgroup (*p* = 0.395)

**Fig. 3:**
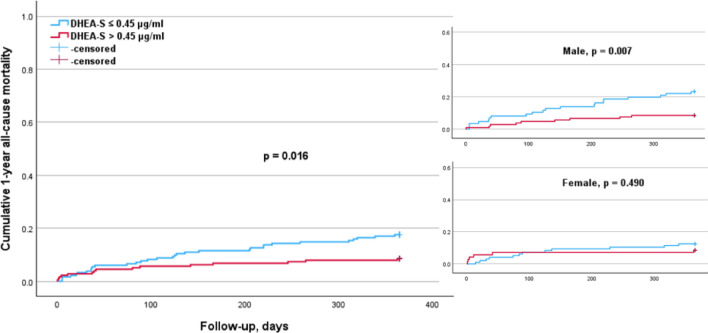
Kaplan–Meier survival analysis of 1-year all-cause mortality according to DHEA-S levels with a cut-off value of 0.45 µg/ml for the entire cohort, as well as for males and females. The analysis shows significant differences in the overall cohort (*p* = 0.016). This was significant in the male (*p* = 0.007), but not in the female subgroup (*p* = 0.490)

**Fig. 4:**
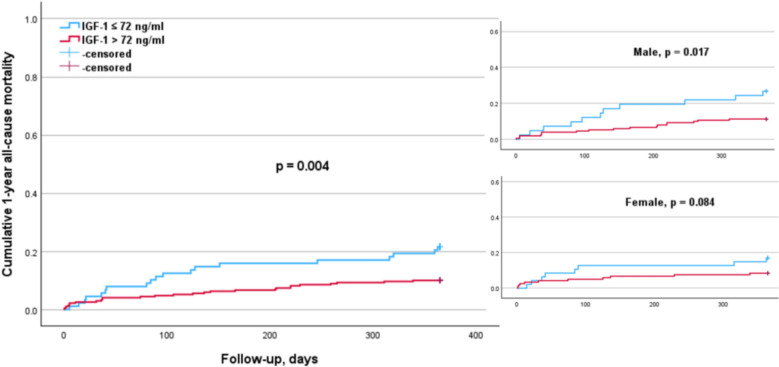
Kaplan–Meier survival analysis of 1-year all-cause mortality according to IGF-1 levels with a cut-off value of 72 ng/ml for the entire cohort, as well as for males and females. The analysis shows significant differences in the overall cohort (*p* = 0.004). This was significant in the male (*p* = 0.017), but not in the female subgroup (*p* = 0.084)

**Fig. 5:**
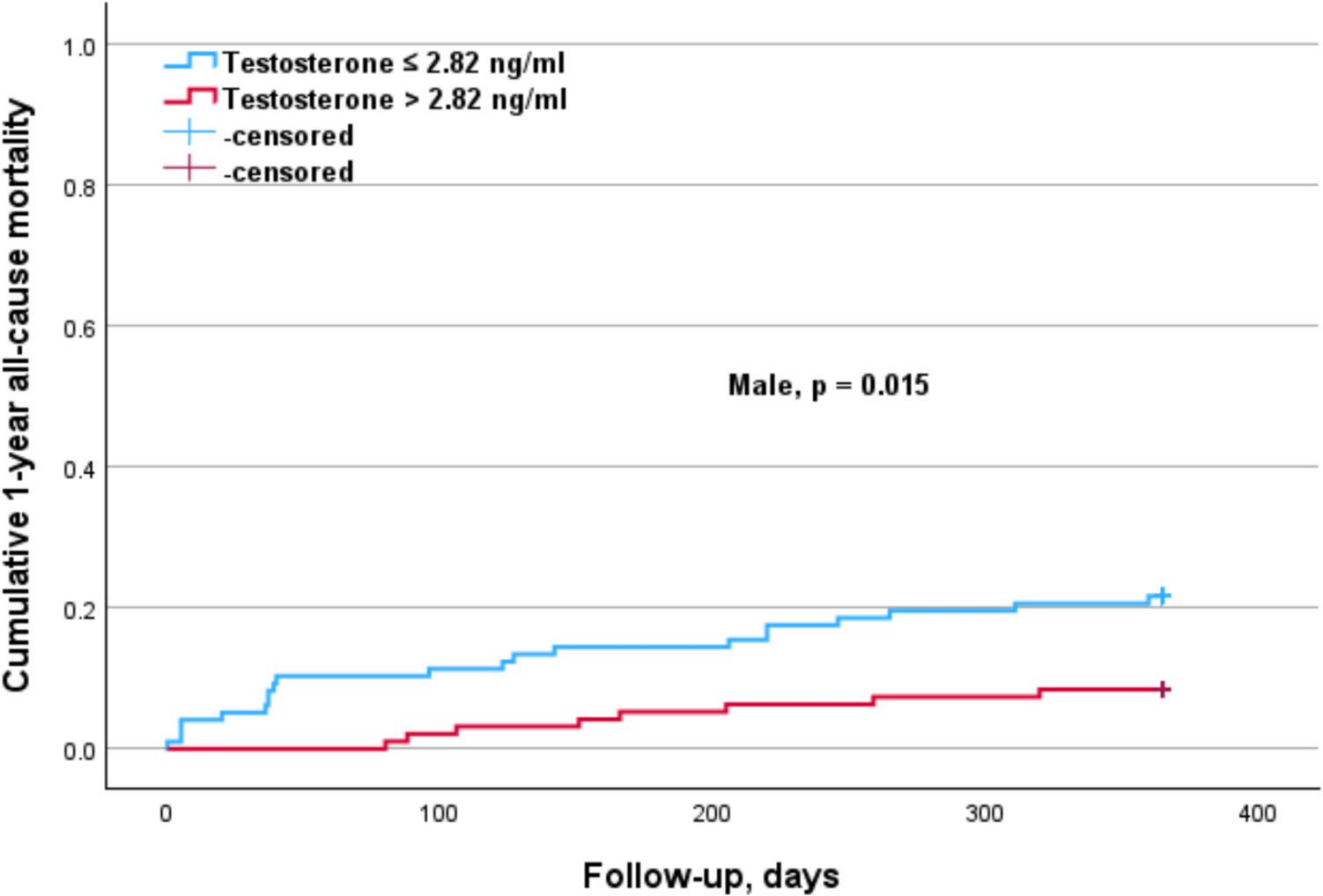
Kaplan–Meier survival analysis of 1-year all-cause mortality according to testosterone levels with a cut-off value of 2.82 ng/ml for males. The analysis shows significant differences (*p* = 0.015)

The subgroup analysis according to sex (Table 4) indicated that elevated cortisol levels (p = 0.011) were significantly associated with a higher 1-year mortality risk in males, but not in females (Fig. 1). Conversely, higher PTH levels were significantly associated with an increased 1-year mortality risk in females (p = 0.012) but not in males (Fig. 2). The elevated DHEA-S levels (p = 0.007) and IGF-1 levels (p = 0.017) were significantly associated with a lower 1-year mortality risk in males, but not in females (Figs. 3, 4). Regarding sex hormones, we found that decreased levels of testosterone below 2.82 ng/ml were associated with a lower 1-year all-cause mortality risk (p = 0.015) in males (Fig. 5).

**Table 4:** Cox regression analysis for 1-year mortality in accordance with hormonal cut-offs and sex-related differences

	Female		Male	
Hormones	HR (95% CI)	*p* value	HR (95% CI)	*p* value
Parathormone > 75 pg/ml	4.94 (1.43–17.08)	0.012	1.38 (0.66–2.88)	0.398
DHEA-S > 0.45 µg/ml	0.71 (0.27–1.89)	0.490	0.34 (0.15–0.74)	0.007
IGF-1 > 72 ng/ml	0.44 (0.17–1.12)	0.084	0.40 (0.19–0.85)	0.017
Cortisol > 8.4 µg/dl	2.12 (0.76–5.95)	0.154	2.66 (1.26–5.64)	0.011
Estradiol > 13 pg/ml	1.62	0.397	1.23	0.657
Progesteron > 0.05 ng/ml	1.65	0.315	1.02	0.953
Testosteron > 2.82 ng/ml	–	–	0.36 (0.16–0.82)	0.015

The 95% confidence interval (CI) for the hazard ratio (HR) was only reported when the *p* value was significant (*p* < 0.05)

## Discussion

This study evaluated sex-related hormonal variances and their impact on outcome in TAVR patients and as expected, we found no significant difference with regard to one-year all-cause mortality rates between sexes. However, we found some relevant sex-related differences, which are as follows:

- Higher cortisol and PTH levels were associated with an increased risk of 1-year mortality, while higher DHEA-S and IGF-1 levels were associated with a decreased risk of 1-year mortality.
- These associations for (cortisol, DHEA-S and IGF-1) were particularly pronounced in male patients, but not in female. Conversely, higher PTH levels were significantly associated with an increased mortality risk in females, but not in males.
- Higher testosterone levels in male patients were associated with a decreased risk of 1-year all-cause mortality.

### Cortisol

Cortisol has long been shown to induce valve calcification in in vivo models [13, 14]. Previous research showed that patients with untreated overt Cushing's Syndrome and mild autonomous cortisol secretion present with a high prevalence of cardiovascular risk factors and increased mortality [15]. Multiple studies have emphasized that excessive cortisol levels are correlated with an increased risk of cardiovascular events, namely acute coronary syndromes, arrhythmias, sudden cardiac death, and stroke [16]. An international, retrospective cohort study including 4374 patients with adrenal incidentalomas and autonomous cortisol secretion showed age- and sex-dependent disparities in mortality, particularly in women younger than 65 years [17]. Our study showed that higher levels of cortisol are associated with an increased risk of 1-year all-cause mortality in AS patients with a median age of 80 years undergoing TAVR. However, although women had statistically significant higher cortisol levels than men, the subanalysis revealed that this association with mortality was only visible in males, supporting the hypothesis that cortisol might have sex-dependent effects on the cardiovascular system.

### PTH

Increased levels of PTH were found to correlate with the progression of aortic valve calcification [18, 19]*. Vadana *et al*.* demonstrated that PTH induces dysfunction in valvular endothelial cells, which in turn stimulates VICs to differentiate into a pro-osteogenic pathological phenotype related to the calcification process [20]. Furthermore, recent studies have shown that higher serum PTH concentrations may lead to cardiovascular disease and may also increase the risk of cardiovascular mortality, mainly by contributing to atherosclerosis and subsequent cardiac events [21, 22]. *El Hilali *et al*.* demonstrated in a longitudinal aging study in the Netherlands on 1317 patients (65–85 years) that men with high PTH concentrations had a higher risk of cardiovascular mortality (HR 3.22; 95% CI: 1.40–7.42) [23]. Similarly, our study results showed that higher PTH levels were significantly associated with an increased mortality risk in females but not in males. These findings suggest a sex-dependent association with mortality risk, which requires further clarification to understand the underlying mechanisms.

### DHEA-S

A recent systematic review of twenty-five studies involving a total of 92,489 patients, examined the association between DHEA-S levels (either on admission or at discharge) and cardiovascular disease outcomes. The review suggested that patients with cardiovascular disease, who have lower DHEA-S levels, may have a poorer prognosis than those with higher DHEA-S levels [24]. A more recent meta-analysis also found that low DHEA-S was associated with an increased risk of total mortality in both men and women, although the association in women did not reach statistical significance [25]. Moreover, previous research suggested a U-shaped association in women [25, 26]. Comparable results could be found in the present study; higher DHEA-S levels were associated with decreased rates of 1-year all-cause mortality, particularly pronounced in male patients.

### IGF-1

IGF-1 deficiency is associated with an increased risk of cardiovascular disease, whereas the cardiac activation of the IGF-1 receptor protects against the detrimental effects of a high-fat diet and myocardial infarction [27]. *Esberg *et al*.* showed that the cardiac survival factor IGF-1 elicited an inhibition of nitric oxide synthase (NOS) activity in left ventricular myocytes from male, but not female, animals. These results suggested that the disparate NOS levels and their response to the cardiac hormone IGF-1 in left ventricular myocytes may contribute to sex-related differences in myocardial mechanical function [28]. Similarly, our analysis revealed that higher IGF-1 levels were associated with a decreased risk of 1-year mortality in male patients with severe AS undergoing TAVR, but not in female patients.

### Testosterone

The association between increased levels of serum testosterone and an increased risk of AS, independent of cardiovascular risk factors, was recently described in a prospective cohort study with 2682 men [29]. A recent systematic literature review on prospective cohort studies, which examined the association of testosterone with all-cause mortality and cardiovascular mortality with at least 5 years of follow-up, showed that men with low testosterone had an increased risk of all-cause mortality and cardiovascular mortality [30]. Our observation aligns with this review, showing that higher testosterone levels in males were associated with a decreased risk of 1-year mortality in TAVR patients.

### Limitations

The retrospective design inherently introduces potential biases related to data collection and patient selection. Additionally, the study was conducted at a single center, which may limit the generalizability of the results to other populations. Although the sample size of 361 patients is relatively substantial, it may still be insufficient to detect subtle hormonal differences and their impact on outcomes, particularly in subgroup analyses. Although the cohort was comparable in terms of risk profiles for this elderly patient population, the study involved multiple testing, introducing an inherent risk of Type I error due to multiple comparisons, which could affect the robustness of the findings.

## Conclusion

Sex-specific hormonal differences significantly impact the prognosis of severe AS patients undergoing TAVR. Elevated cortisol levels and decreased DHEA-S and IGF-1 levels in males, as well as higher levels of PTH in females, were associated with an increased mortality risk after TAVR. These hormones have the potential to serve as biomarkers for risk stratification, further highlighting the protective role of DHEA-S and IGF-1. Their consideration in future therapeutic approaches, particularly for the elderly population, is warranted. However, these findings are still hypothesis-generating; larger and prospective studies are needed to confirm potential clinical applications.

## Abbreviations

AS: Aortic valve stenosis
TAVR: Transcatheter Aortic Valve Replacement
C-AS: Critical aortic valve stenosis
SHBG: Sex hormone-binding globulin
IGF-1: Insulin-like growth factor 1
DHEAs: Dehydroepiandrosterone sulfate
AVA: Aortic valve area
